# Predicting the Lateral Load Carrying Capacity of Reinforced Concrete Rectangular Columns: Gene Expression Programming

**DOI:** 10.3390/ma15072673

**Published:** 2022-04-05

**Authors:** Raheel Asghar, Muhammad Faisal Javed, Raid Alrowais, Alamgir Khalil, Abdeliazim Mustafa Mohamed, Abdullah Mohamed, Nikolai Ivanovich Vatin

**Affiliations:** 1 Department of Civil Engineering, Abbottabad Campus, COMSATS University Islamabad, Abbottabad 22060, Pakistan; raheelasghar@cuiatd.edu.pk; 2 Department of Civil Engineering, Jouf University, Sakaka, Al-Jouf 72388, Saudi Arabia; rnalrowais@ju.edu.sa; 3 Department of Civil Engineering, University of Engineering and Technology, Peshawar 25120, Pakistan; alamgirkhalil@uetpeshawar.edu.pk; 4 Department of Civil Engineering, College of Engineering, Prince Sattam Bin Abdulaziz University, Alkharj 16273, Saudi Arabia; a.bilal@psau.edu.sa; 5 Building and Construction Technology Department, Bayan College of Science and Technology, Khartoum 210, Sudan; 6 Research Centre, Future University in Egypt, New Cairo 11835, Egypt; mohamed.a@fue.edu.eg; 7 Peter the Great St. Petersburg Polytechnic University, 195291 St. Petersburg, Russia; vatin@mail.ru

**Keywords:** reinforced concrete columns, lateral load carrying capacity, bearing capacity, flexural capacity, shear capacity, gene expression programming

## Abstract

This research presents a novel approach of artificial intelligence (AI) based gene expression programming (GEP) for predicting the lateral load carrying capacity of RC rectangular columns when subjected to earthquake loading. To achieve the desired research objective, an experimental database assembled by the Pacific Earthquake Engineering Research (PEER) center consisting of 250 cyclic tested samples of RC rectangular columns was employed. Seven input variables of these column samples were utilized to develop the coveted analytical models against the established capacity outputs. The selection of these input variables was based on the linear regression and cosine amplitude method. Based on the GEP modelling results, two analytical models were proposed for computing the flexural and shear capacity of RC rectangular columns. The performance of both these models was evaluated based on the four key fitness indicators, i.e., coefficient of determination (*R*^2^), root mean squared error (*RMSE*), mean absolute error (*MAE*), and root relative squared error (*RRSE*). From the performance evaluation results of these models, *R*^2^, *RMSE*, *MAE*, and *RRSE* were found to be 0.96, 53.41, 38.12, and 0.20, respectively, for the flexural capacity model, and 0.95, 39.47, 28.77, and 0.22, respectively, for the shear capacity model. In addition to these fitness criteria, the performance of the proposed models was also assessed by making a comparison with the American design code of concrete structures ACI 318-19. The ACI model reported *R*^2^, *RMSE*, *MAE*, and *RRSE* to be 0.88, 101.86, 51.74, and 0.39, respectively, for flexural capacity, and 0.87, 238.74, 183.66, and 1.35, respectively, for shear capacity outputs. The comparison depicted a better performance and higher accuracy of the proposed models as compared to that of ACI 318-19.

## 1. Introduction

### 1.1. Background

Columns are the primary structural elements intended to transfer the load of the overlying structure to the foundations underneath through axial compression [1]. Based on the material, they can be classified into several types. However, reinforced concrete (RC) columns are widely used in buildings and bridge structures. They are generally considered as the most important RC frame members of these structures in terms of their seismic response [2,3]; this is because most of the recent structural failures during earthquakes are reported to occur because of poor column performances [4]. A failure normally triggered through the columns progressively results in the global failure of the overall structure. The modern generation of earthquake engineering aims to equip structural engineers with advanced techniques to design structures based on their performance rather than on conventional strength-based methods [5,6,7,8]. To effectively apply this approach for the seismic design of RC rectangular columns, failure modes and ultimate load-carrying capacities are required to be determined [9]. Enough literature is already available on the failure mode classification of RC columns. According to Acun and Sucuoglu [10], failure in columns occurs because of insufficient strength or deformation capacity. Based on this principle, RC column failures can be classified into three categories [11], i.e., shear failure, flexural failure, and flexural-shear failure. Shear failure occurs in a brittle manner with a sudden loss of load carrying capacity before the yielding of longitudinal reinforcement, and therefore also termed as brittle shear failure [12]. Flexural failure occurs in a ductile fashion, with the column enduring a large deformation without any significant loss of load-carrying capacity [12]. This type of failure is also known as a ductile flexural failure. An intermediate failure stage exists between pure shear and flexural failure, i.e., flexural-shear failure. In this type of failure, columns usually suffer material damage or flexural yielding just before experiencing pure shear failure [12]. This is also termed as ductile shear failure.

Many researchers have worked on quantifying these failure modes of RC columns. Some have presented pure analytical techniques for classifying these failure modes based on either geometrical or strength parameters, whereas others have presented mixed analytical-experimental approaches. According to Feng et al. [13], the failure mode classification is normally based on two simple indices, i.e., aspect ratio (*A_r_*) and shear demand to the capacity ratio. If *A_r_* (i.e., the ratio of column length to the cross-sectional height) is less than 2, the column failure would be categorized as a pure shear failure, whereas if it is greater than 4, the failure would be classified as a pure flexural failure. If 2 ≤ *A_r_* ≤ 4, the failure is said to be a flexural-shear failure. Similarly, if the shear demand to capacity ratio is greater than 1, the column would suffer pure shear failure, whereas if it is less than 0.7, the failure would be a pure flexural failure. However, if the shear demand to capacity ratio is between 0.7 and 1, combined flexural-shear failure is expected to occur for the given RC column [13]. 

Once the failure mode is classified, the next step is determining the lateral load-carrying capacity (i.e., bearing capacity) of the RC columns. This can be defined as the maximum lateral load taken by the column before undergoing failure during seismic events. The bearing capacity of an RC column further depends on its flexural and shear capacities, the minimum of which is regarded as the bearing capacity of the overall column. The flexural capacity is normally based on the plane section assumptions and can be determined accurately using the famous flexural formula (f = MY/I). However, the determination of shear capacity is relatively complicated because of multiple influencing factors and complex shear transfer mechanisms. Many experimental and finite element studies have predicted both these capacities of RC columns with higher accuracy [1,11,14], but these traditional capacity determination techniques are costly, time-consuming, and laborious. The solution to the problem lies in the development of analytical models [13,15,16,17,18,19,20]. Up till now, many empirical models have been developed for the prediction of bearing capacity of RC columns, e.g., failure mode and bearing capacity prediction model [13], strut and tie model [21,22], modified compression field theory model [23,24], softened truss model [25,26], damage model [27,28], etc. However, most of these models are based on simple regression analysis incorporating limited experimental datasets [13]. Therefore, their applicability in a broader aspect is always questionable. Moreover, the models with relatively larger datasets have considered only a few parameters in their model development. Therefore, their accuracy is also not ideal. Artificial intelligence (AI) techniques are generally recommended to overcome these issues using relatively larger databases developed over the past few decades [29]. AI is a “black box” that relates the inputs and outputs in a simplified manner while avoiding complex mathematical derivations. It has better performance as compared to the traditional analytical models and therefore can be employed to predict the bearing capacity of RC columns quickly, accurately, and robustly.

The term AI was first introduced in 1956 [30] to represent the ability of a machine to imitate intelligent human behaviour in order to solve complicated problems based on human-inspired algorithms [31]. However, its use was limited then in the civil and structural engineering community because of the lack of innovative techniques and advanced computer technology. The recent progress in AI techniques and modern computer systems has enhanced the interest of structural engineers in developing precise and consistent models to solve complex structural problems [32]. AI procedures are generally based on machine learning (ML), pattern recognition (PR) and deep learning (DL), which further consist of artificial neural networks (ANNs), fuzzy logic, genetic programming (GP), etc. [16,17,31,33,34,35,36,37,38,39,40,41,42,43,44,45,46,47,48,49,50]. The application of these AI techniques in structural engineering are traced back to the early 1980s, where they were first used in the compliance checking of design codes [51,52] and expert interactive design of concrete columns (EIDOCC) [53,54]. In addition to these basic works, an AI technique, i.e., ML, was also applied to predict the location and extent of the damage to various structural systems subjected to varying loading conditions [55,56,57]. Multiple AI techniques, including ANN, logistic and linear regressors, lasso and supper vector machines (SVMs), have been adopted to predict the fragility behaviour of RC structural systems [58,59,60,61]. Some recent works have also employed AI techniques to predict the shear strength of RC beams [62], shear strength of RC joints [63], punching shear capacity of RC slabs [64], failure mode and capacity prediction of RC columns, etc. [12,65,66]. However, the methodology adopted in all these works requires a bulk of memory to accommodate a large number of hidden neurons involved in the model development. Moreover, using these techniques, it is also difficult to represent the relationship between inputs and outputs in a practically simplified form, which requires the researchers and other stakeholders to shift towards more efficient, simple, and practically applicable techniques.

**Figure 1:**
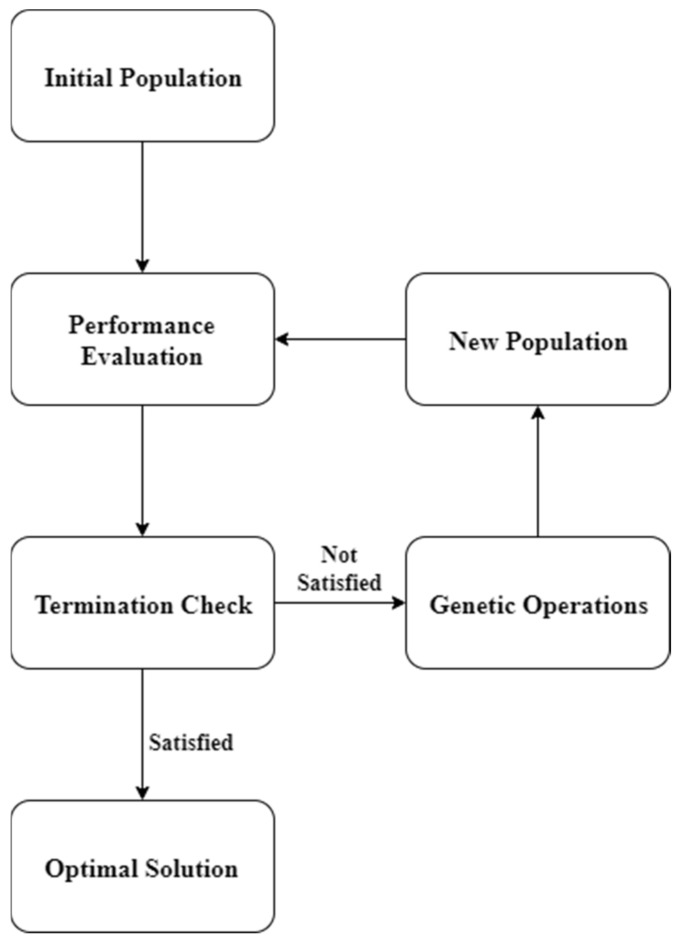
Working Procedure of GP Algorithms.

Another AI method, i.e., GP, provides an effective solution to the above-discussed practicality issue. This was first introduced by Cramer in 1985 [67] and further improved by Koza in 1992 [68]. It is an evolutionary AI algorithm intended to produce high-quality solutions to complicated problems through analogues to computer-based genetic operations, e.g., deletion, reproduction, duplication, mutation, and crossover. It is a domain-independent technique based on Darwin’s evolutionary theory [69]. GP starts with the given initial population of programs consisting of multiple functions and terminals (Figure 1). The functions describe the standard procedure to execute the program, whereas terminals outline the arguments for each function [70,71]. The functions may include arithmetical operations, program manoeuvres, and mathematical equations. The performance of a certain program is evaluated based on its effectiveness in explaining the output while exploiting the given inputs. Evaluating the initial population, worst performing programs are deleted, whereas a new generation of programs is created based on the top performing programs through the operations of reproduction, mutation, duplication, and crossover [68]. The mutation involves replacing an arbitrary part of a program with an alternative part randomly picked from another program of the existing population. In addition to the reproduction of the programs through mutation and crossover, some programs are simply copied from the previous generation to the new generation through a process known as duplication. These processes eventually result in the production of a new generation of programs having a better fitness than their parent population [68]. This loop of genetic operations is repeated again and again until an ultimate termination condition is reached. Some common termination conditions of GP may include maximum number of iterations, maximum allowable computational time, minimum level of difference between the populations of successive iterations or a solution satisfying the set criteria. In case either of these conditions is reached, the program is terminated to provide a final solution [68]. 

Recently, GP has been successfully used as an automated problem-solving technique in many practical applications, e.g., engineering design, traffic routing, curve fitting, data modelling, failure classification, capacity prediction, symbolic regression, etc. [72]. However, along with all the positive utilities it offers, there are certain limitations associated with it. It is computationally very expensive in the case of high dimensional real-world problems which require a huge amount of time to complete simulation to get the optimum results [73]. It also tends to destructive mutation, i.e., once the best fit chromosome is generated, it can be reconstructed into a lesser fit chromosome of the new generation, reducing its overall efficiency [73]. The effectiveness of GP is always a matter of debate in the case of decision problems, where there is the least probability for the convergence of a solution [73]. It can also sometimes result in an exponential increase in the population size during mutation and crossover operations, making this technique difficult to apply to complex design problems [73]. Another drawback of GP lies in its inability to have an independent genome. Therefore, its nonlinear structure has to act both as a genotype and phenotype [74], which makes it incapable of producing simple and robust expressions. To successfully deal with all these limitations, an extension of GP, i.e., gene expression programming (GEP), is often recommended. GEP further improves the effectiveness of this technique by making the model adaptive to different conditions, like living organisms. It uses the simple chromosomes of defined length to encode a computer program for the given model [74]. GEP has the ability to describe the output by a simple mathematical equation, which can be recommended for practical purposes with higher prediction accuracy [75]. Recently, GEP has been considered an alternative to the conventional analytical methodologies, particularly in civil and structural engineering [76].

Therefore, this research aims to predict the lateral load carrying capacity of RC rectangular columns subjected to earthquake loading utilizing GEP. Moreover, as according to the authors’ knowledge, no equation has been proposed in the past research articles until now to directly predict the lateral load carrying capacity of RC columns, this research also aims to provide simplified empirical relationships for future design purposes while assuring the universal nature of the proposed model. It is further extended to provide a comparison on the lateral load carrying capacity of RC columns as recommended by the proposed model and international design codes.

### 1.2. Gene Expression Programming

GEP was proposed by Candida Ferreira [77] in 2001 as an extension of GP. It belongs to the family of evolutionary AI algorithms that create complex tree structure computer programs, often known as parse trees. Multiple parse trees exist in each chromosome of these computer programs, which are usually created by the multigenic system of GEP. Therefore, they are also known as expression trees [78,79]. These computer programs are supposed to explore the environment and adapt to the given conditions by altering their sizes, shapes, and composition. The computer programs are generally encoded using simple linear chromosomes of fixed length as inherited from genetic algorithms (GA), resembling the genetic model of living organisms. GEP incorporates these linear chromosomes as genotype, and the parse trees as phenotype of their complex system of genomes and phenomes [79]. The genomes are intended to keep and transfer genetic information, whereas phenomes are responsible for exploring and adapting the programs to the given environment. Despite their fixed sizes, genomes are used to encode the expression trees of varying sizes and shapes, allowing the adaptation and evolution of phenomes to occur smoothly. An expression tree generally consists of a phenome expression and the linear strings of GEP genes [79]. These linear string genes are often known as k-expressions which can be read directly from top to bottom and left to the right in the expression tree structure of GEP. The k-expressions are normally used to represent the region of genes that are being expressed, which in turn epitomises the validity of computer programs and expressions. GEP genes can be further expressed as a function of two domains, i.e., a head and a tail [77]. The head is normally used to encode all the input variables and functions chosen to get the required solution, whereas the tail provides a pool of terminals to ensure the error-free nature of the GEP programs. Following the gene structure of living organisms, GEP genes are also associated with the homeotic genes, which controls the interaction between different main program modules [79]. Homeotic genes possess an identical structure to the normal GEP genes as they are the product of the same process. They are also comprised of a head and a tail domain. The only difference is that they contain linking functions and special terminals called genic terminals. The cellular system in GEP allows the unrestricted evolution of linking functions as well as the recursive use of encoded programs. The head-tail domains of GEP and homeotic genes are the fundamental units of all GEP algorithms [79]. However, GEP also tries to explore additional genes with an extra domain. This extra domain usually encodes random constants that are persistently fined tuned by the program to arrive at optimum solution.

**Figure 2:**
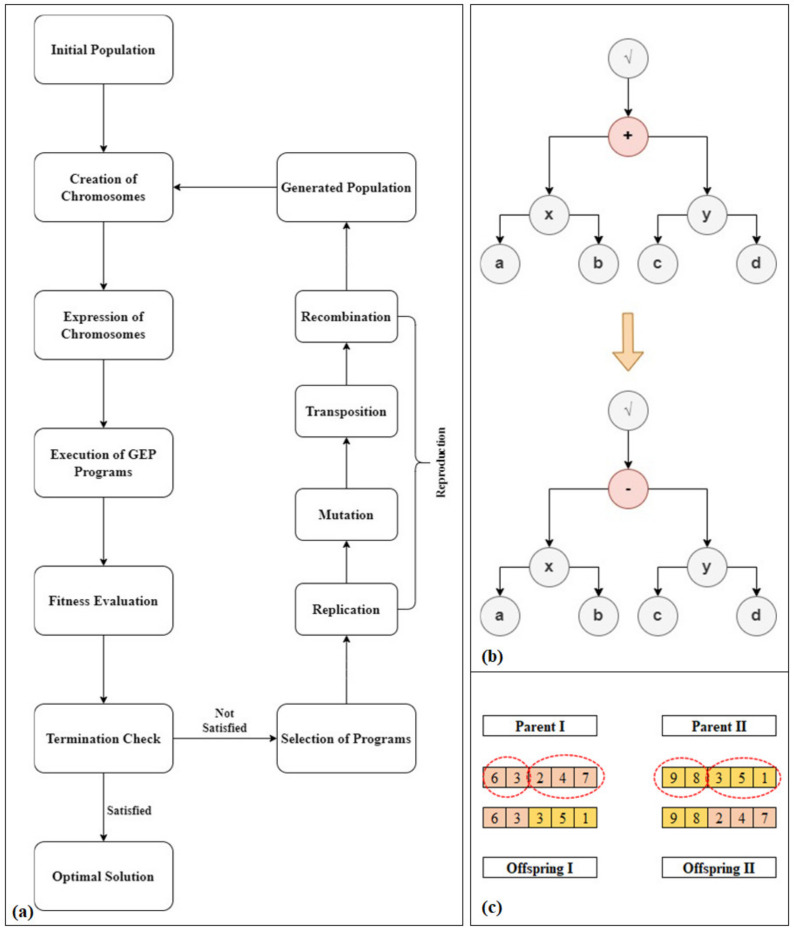
(**a**) GEP Algorithm (**b**) Mutation Process (**c**) Crossover Process.

The basic GEP algorithm involves four fundamental steps, i.e., the creation of initial random population, execution of GEP programs, verification of termination conditions and the generation of subsequent populations or the representation of the final optimum solution in the form of a simple mathematical expression (Figure 2). The initial random population of GEP programs is usually created with the help of functions and terminal sets. GEP only requires the population units to be expressed in the form of simple linear chromosomes of fixed length without wondering about their structural reliability [79]. This is because GEP expressions always result in syntactically sound programs. After creating the initial random population, all the GEP programs are executed according to predefined fitness functions. These fitness functions normally depend on the type of problem under consideration. Based on prediction outputs, the overall GEP problems can be classified into three main categories, i.e., the regression problems, classification problems and Boolean logic problems [79]. For the regression problems, the output is usually a numeric value. Therefore, the fitness function for the evaluation of their model performance is based on the error between the model output and actual output. The most frequently used fitness functions for regression problems include the coefficient of determination (*R*^2^), root mean squared error (*RMSE*), mean absolute error (*MAE*), mean absolute percentage error (MAPE), number of loop iterations, etc. [79]. 

The output of classification and Boolean logic problems generally cannot be expressed in the form of numeric values; therefore, their performance evaluation functions are based on a matrix counting the number of correct and incorrect predictions, often known as a confusion matrix. Some common fitness functions based on this confusion matrix include sensitivity, precision, Jaccard similarity, F-measure, etc. [79]. These fitness functions are quite sophisticated and are adequate for efficient solution of most of the classification and Boolean logic problems. However, any complexity encountered while dealing with these problems can be solved effectively by introducing an additional fitness function capable of exploring the overall model structure, distribution of outputs and classifier margins [79]. Once the GEP programs are executed, their performance is evaluated based on the already defined above-discussed fitness functions. If the evaluation results satisfy the termination criteria, the GEP loop algorithm is terminated, and the final optimum solution is presented in the form of a simple mathematical expression. However, if the termination criteria are not satisfied, the best-fit programs are selected to reproduce the subsequent population of GEP programs, and the loop is repeated [77]. The reproduction process of the GEP algorithm involves the four basic genetic operations, i.e., replication, mutation, recombination, and transposition. During replication, all the genomes of the best performing programs of the previous population are copied to the new population. The sub-structural elements of these genomes are then replaced with one another during the mutation process of genetic reproduction [77]. After the basic operations of replication and mutation, the new chromosomes of the next generation are produced by combining different parts of the parent chromosomes. To ensure the soundness and reliability of the generated programs, a transposition insertion sequence is introduced in the heads of genes while preserving the chromosome length and gene structure [79]. This loop algorithm of GEP is repeated again and again until an optimum solution is obtained (Figure 2).

A significant characteristic of GEP is that only the genome is transferred to the successive population of computer programs instead of the global parent structure, which enhances the overall algorithm efficiency [77]. There also exists a direct relationship between the chromosomes and corresponding functions or terminals which makes the genetic variation of GEP simple and easy to understand [32]. Another advantage of GEP lies in its ability to provide the optimum solution with simple mathematical equations [17]. These equations can be easily manipulated in practical works, especially in civil and structural engineering. Considering all these positive features, this research aims to employ GEP to predict the lateral load carrying capacity of RC rectangular columns subjected to earthquake loading.

## 2. Methods

The development of an analytical model for predicting the bearing capacity of RC rectangular columns involves three fundamental steps i.e., the preparation of a comprehensive universal database, selection of model parameters, and the implementation of the GEP algorithm. According to Mundfrom et al. [80], the minimum sample size in the database must be 3–20 times the involved parameters for general engineering problems. However, it is still a controversial topic and is often decided according to logical judgment criteria [13]. The selection of parameters for the model development can be based on linear regression or other simpler analysis determining the relationship between two or more variables. Once an integrated and comprehensive database is established, the GEP algorithm can be implemented to obtain the required analytical model for the given problem.

### 2.1. Database

An inclusive structural performance database assembled by the PEER centre [29] was adopted to develop an analytical model for predicting RC rectangular columns’ bearing capacity. This was prepared by the researchers based on the experimental work at the National Institute of Standards and Technology. It contains all the data required to evaluate the seismic performance of RC columns. The overall information provided in the database can be divided into two main categories, i.e., the key column properties (e.g., material properties, geometric properties, confinement details and test configuration) and experimental test results (e.g., failure modes, bearing capacities, force–displacement relationship, axial load effect, and damage pattern). The database is currently available on the worldwide websites of the University of Washington [81] and the PEER centre [82]. However, PEER website provides additional information about the experimental work, i.e., structural drawings, testing images, etc., compared to the University of Washington’s website.

The distribution of some important column properties, i.e., depth, aspect ratio, axial load ratio, longitudinal reinforcement ratio and transverse reinforcement ratio, was examined to evaluate the comprehensiveness and universal nature of the model database (Table 1). From the detailed statistical analysis, the database was found to be normally distributed about a mean value of 319 mm with respect to depth, 3.6 with respect to the aspect ratio, and 2.39% with respect to longitudinal reinforcement ratio. However, it was found to be skewed more towards the lesser values of aspect ratio, longitudinal reinforcement ratio and axial load ratio. The analysis has also shown that approximately 80% of the columns have depths ranging between 200 and 500 mm, whereas 65% have an axial load ratio of between 0 and 0.3. The database was also found to be weighted around a mean value of 2% of the transverse reinforcement ratio. However, no characterizable distribution was observed. Analysing the distribution characteristics of all the above discussed column properties, the database can be said to possess population samples from one extreme to the other following a specific distribution, more often a normal distribution, which indicates its inclusiveness and universality.

### 2.2. Parametric Selection

The structural performance database assembled by the PEER centre provides more than 40 parameters describing all aspects of the seismic response of RC rectangular columns. The selection of output variables was made conferring to the set objective (i.e., determining the bearing capacity of RC rectangular columns) according to which flexural capacity and shear capacity outputs were selected. However, for selecting input variables against the established outputs, a detailed stepwise procedure was adopted. A linear regression analysis was performed using Microsoft Excel while considering all the available input variables in the first step. The analysis results showed a strong relationship between the inputs and outputs with a coefficient of determination (*R*^2^) of 0.9408 for flexural capacity output and 0.9339 for shear capacity output. However, the number of variables employed in the regression analysis was found to be too much to be expressed by a simple mathematical expression. Therefore, reducing the number of inputs was decided while ensuring minimum damage to the statistical relationship between the inputs and outputs. To reduce the number of input parameters, all the groups of similar variables were replaced by their corresponding single universal variable (e.g., instead of width and height, area parameter was used, which is the combined/universal variable for the preceding two variables), based on the fundamental principles of civil and structural engineering. This utilization of a single universal variable instead of a number of default variables reduced the number of input parameters to 12, almost one-third of their initial quantity, i.e., 35. Linear regression analysis was performed again to assess the damage caused by the reduction of input variables to the statistical relationship between the inputs and outputs. However, the analytical results showed no significant impairment as the coefficient of determination was found to be 0.8912 in the case of flexural capacity output, and 0.8784 in the case of shear capacity output.

A combined approach of the linear regression and cosine amplitude methods was employed to further reduce the number of input variables for the model development. Linear regression along with analysis of variance (ANOVA) was performed in Microsoft Excel to calculate the *p*-value, whereas the cosine amplitude method was used to compute the relationship coefficient *R* for each parameter (Table 2). The *R*-value was obtained according to Equation (1), where *X_1_* and *X_2_* represent the parameters, whose relationship is to be determined, and *“m”* epitomises the sample size. For the acceptability of each of the above discussed modified parameters, a maximum threshold of 0.05 for the *p*-value, and a minimum threshold of 0.75 for the *R*-value, were established. Any parameter satisfying either of these criteria for the flexural or shear capacity output was selected as the model input for predicting the bearing capacity of RC rectangular columns. This combined approach of linear regression and cosine amplitude method reduced the number of input variables to approximately half of their previous quantity, i.e., seven (Table 3). Linear regression analysis was performed again to evaluate the damage caused by this reduction of input variables to the statistical relationship between the inputs and outputs. However, the analysis results showed no substantial loss as the coefficient of determination was 0.8896 for flexural capacity output, and 0.8760 for shear capacity output, which is similar to that calculated earlier employing 12 variables.

(1) $$R-Value=\frac{\left|\sum_{k=1}^{m}X_{1k\;}.\;\;X_{2k}\right|}{\sqrt{\left(\sum_{k=1}^{m}X_{1k}^{2}\right)\;\;\left(\sum_{k=1}^{m}X_{2k}^{2}\right)}},$$

### 2.3. GEP Modelling

The modelling of flexural and shear capacity models for predicting the overall bearing capacity of RC rectangular columns was carried out using an extremely flexible GEP data modelling software, GeneXproTools 5.0. This is an easy and resourceful data mining software especially designed for simple functional regression, logistic regression, classification, time series prediction and logic synthesis. A well-organized Microsoft Excel database consisting of seven input variables against the established outputs was imported into GeneXproTools to initialize the modelling process. For effective and robust model development, all the 250 population samples were regarded as both the training and validation datasets [75,83,84,85]. GeneXproTools exhibits a distinct characteristic where it allows the user to specify the key modelling parameters, i.e., the number of chromosomes, head size, number of genes, linking function, constant per gene, model functions, etc., for the preparation of the predictive model. Exploiting this characteristic of GeneXproTools, multiple GEP models were created using different combinations of modelling parameters. A brief summary of the modelling characteristics of these GEP models is given in Table 4. The performance of all these models was then evaluated based on the four most commonly used fitness measures, i.e., coefficient of determination (*R*^2^), root mean squared error (*RMSE*), mean absolute error (*MAE*), and root relative squared error (*RRSE*). A higher *R*^2^, and lower *RMSE*, *MAE*, and *RRSE*, usually represent a better fit model. There also exists another performance measure, i.e., the correlation coefficient. However, this was ignored because of its insensitivity towards the multiplication and division of outputs to a constant [86]. Based on these performance indicators, the best fitted model was proposed for future practical purposes.

- Coefficient of Determination (*R*^2^)
  (2) $$R^{2}=1-\frac{\sum_{i=1}^{m}{\left(P_{i}-T_{i}\right)}^{2}}{\sum_{i=1}^{m}{\left(P_{i}-\bar{T}\right)}^{2}},$$
- Root Mean Squared Error (*RMSE*)
  (3) $$RMSE=\sqrt{\frac{\sum_{i=1}^{m}{\left(P_{i}-T_{i}\right)}^{2}}{m}},\;\;$$
- Mean Absolute Error (*MAE*)
  (4) $$MAE=\frac{\sum_{i=1}^{m}\left|P_{i}-T_{i}\right|}{m},$$
- Root Relative Squared Error (*RRSE*)
  (5) $$RRSE=\sqrt{\frac{\sum_{i=1}^{m}{\left(P_{i}-T_{i}\right)}^{2}}{\sum_{i=1}^{m}{\left(T_{i}-\bar{T}\right)}^{2}}},$$

Here *P* represents the predicted values, *T* represents the tested values or experimental values, $\bar{T}$ represents the mean tested value, and *m* represents the number of population samples.

## 3. Results

The nominal failure in RC rectangular columns subjected to earthquake loading occurs when either of their shear or flexural capacity is reached. This is because shear strength and flexural strength are the only parameters triggering the failure of RC rectangular columns in seismic conditions. Based on this fact, two types of capacity prediction models, i.e., the flexural capacity model and shear capacity model, are required to explain their overall bearing capacity comprehensively. Based on the results obtained from both these models, the ultimate bearing capacity or lateral load carrying capacity (*V_u_*) of the aforementioned columns can be obtained using Equation (6).

(6) $$V_{u}=\min\left(\frac{M_{F}}{L},V_{S}\right),$$

### 3.1. Flexural Capacity Model

**Figure 3:**
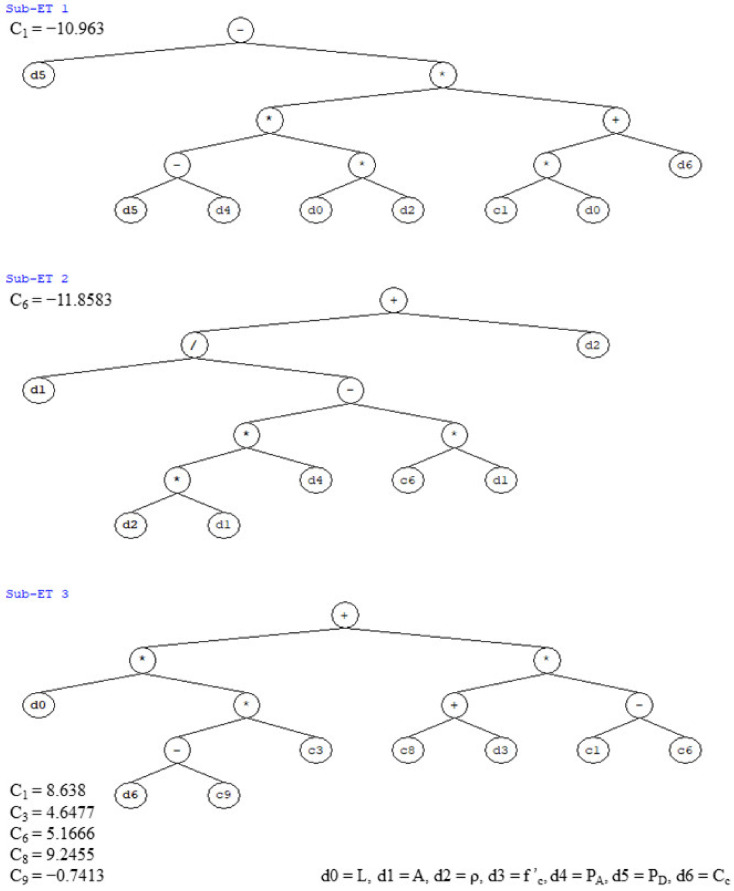
GEP Expression Tree for Flexural Capacity Model.

To develop the analytical model for the prediction of the flexural capacity of RC rectangular columns, multiple GEP models were created in GeneXproTools with different combinations of modelling parameters, a summary of which is shown in Table 4. The performance of all these models was assessed based on the already defined fitness indicators, i.e., *R^2^*, *RMSE*, *MAE*, and *RRSE*. Based on the evaluation results, the best performing GEP model, i.e., the model with the highest value of *R^2^* and least value of *RMSE*, *MAE*, and *RRSE,* was selected and proposed for predicting the flexural capacity of RC rectangular columns for future practical purposes. The GEP expression tree was extracted from the modelling software GeneXproTools as shown in Figure 3, to ensure the effective and easy utilization of the proposed model in real-world problems. This expression tree was then decoded to be presented in the form of simple mathematical expressions as given in Equations (7)–(10). Here, Equation (7) can be used to predict the flexural capacity of RC rectangular columns, the input parameters of which can be calculated using Equations (8)–(10). The rest of the variables used in all these equations are already defined in Table 3. However, attention must be paid to the measuring units.

(7) $$M_{F}=\frac{X_{1}}{X_{2}\;.\;\;X_{3}},$$

(8) $$X_{1}=P_{D}-\left[\left\{\rho L\left(P_{D}-P_{A}\right)\right\}\;.\;\left\{C_{c}-10.963L\right\}\right],$$

(9) $$X_{2}=\rho +\frac{1}{\rho P_{A}+11.8583},$$

(10) $$X_{3}=4.6477L\;\left(C_{c}+0.7413\right)+3.4714{f^{\prime }}_{c}+32.0948,$$

### 3.2. Shear Capacity Model

**Figure 4:**
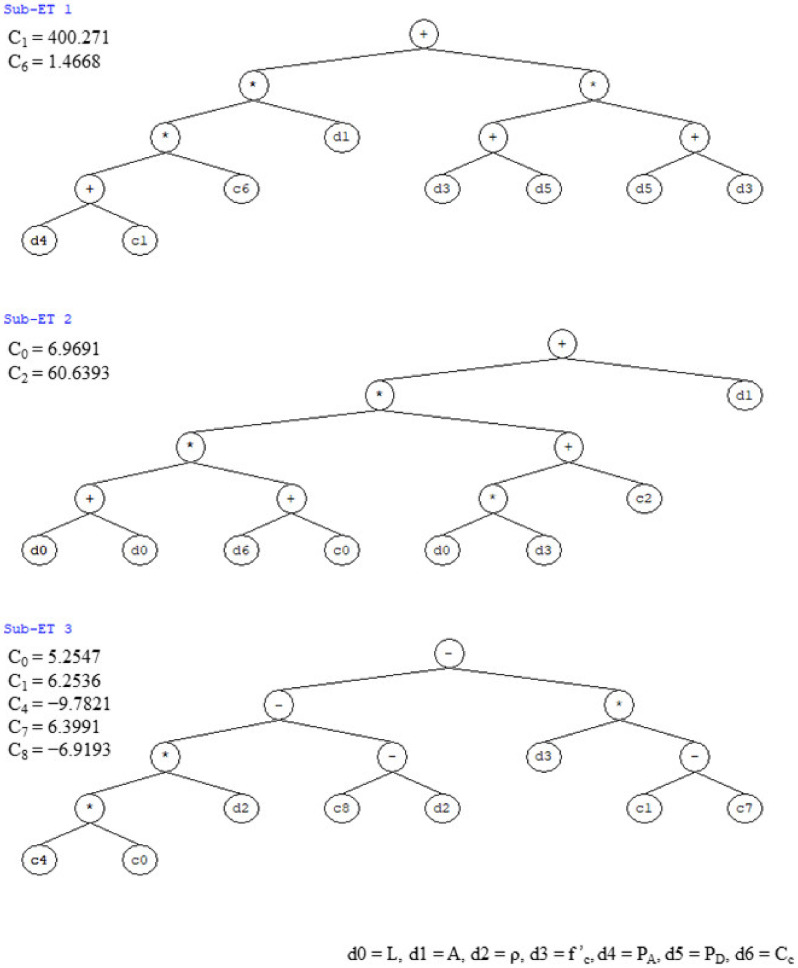
GEP Expression Tree for Shear Capacity Model.

A similar procedure was adopted to develop the analytical model for predicting shear capacity of RC rectangular columns as used for the development of the flexural capacity model. The best performing GEP model from all those shown in Table 4 was selected based on the same performance criteria as described earlier in the flexural capacity model. The expression tree of this model (Figure 4) was extracted from GeneXproTools, which was then decoded and expressed in terms of simplified mathematical expression. Here, Equation (11) was proposed to predict the shear capacity of RC rectangular columns. The parameters involved in this equation can be calculated using Equations (12)–(14).

(11) $$V_{S}=\frac{X_{A}}{X_{B}\;.\;\;X_{C}},$$

(12) $$X_{A}=1.4668A\;\left(P_{A}+400.271\right)+{\left({f^{\prime }}_{c}+P_{D}\right)}^{2},$$

(13) $$X_{B}=A+\left[\left\{2L\;\left(C_{c}+6.9691\right)\right\}\;.\;\;\left\{L{f^{\prime }}_{c}+60.6393\right\}\right],$$

(14) $$X_{C}=0.1455{f^{\prime }}_{c}-50.402\rho +6.9193,$$

### 3.3. Model Validation

**Figure 5:**
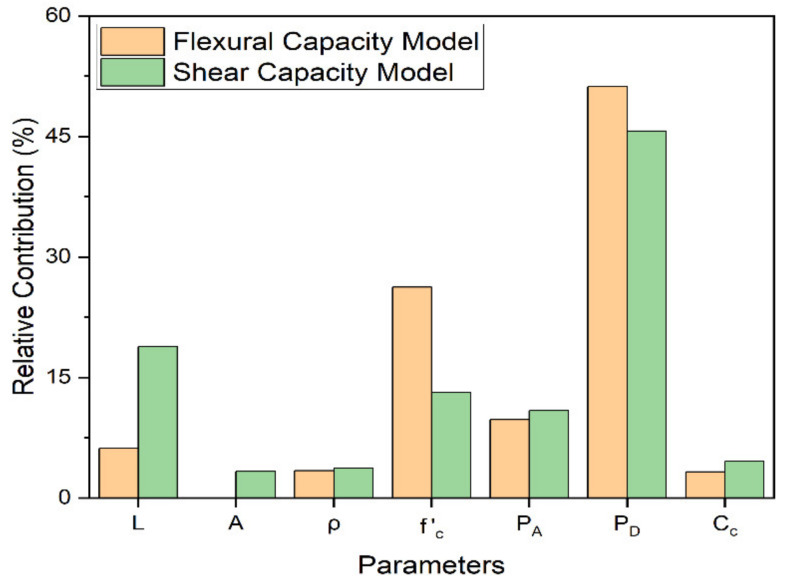
Relative Contribution of Input Parameters to the Model Development.

In AI-based data modelling, it is often recommended to conduct a variety of statistical analyses to ensure the proposed models’ robustness and universality [67]. Two types of such analysis, i.e., the sensitivity analysis and the parametric analysis, were employed in this research to investigate whether the proposed models represent the actual physical phenomenon or not [85]. The sensitivity analysis is normally used to explore input variables’ relative contribution (RLC) towards the overall model development. The mathematical expressions for conducting the sensitivity analysis are given in Equations (15) and (16) [67]. Here, *f_max_ (x_i_)* and *f_min_ (x_i_)* represent the maximum and minimum predicted output corresponding to the *i^th^* input variable in the database while maintaining all other variables at their average value. From the sensitivity analysis results, *P_D_* was found to be the most significant input variable contributing around 50% towards the development of flexural and shear capacity models of RC rectangular columns (Figure 5). The rest of six variables i.e., *L*, *f*^’^ *_c_*, *P_A_*, *ρ*, *C_c_* and *A* cumulatively account for the remaining 50% of the overall model development.

(15) $$RLC\;\left(\%\right)=\frac{N_{i}}{\sum_{i=1}^{n}N_{i}}.100,$$

(16) $$N_{i}=f_{max}\left(x_{i}\right)-f_{min}\left(x_{i}\right),$$

#### 3.3.1. Impact Assessment of Model Influencing Parameters

Parametric analysis was conducted to further validate the efficiency of the proposed models to capture behind-scenes real-world phenomena. In the parametric analysis, the trend of the output variable to a certain change in a specific input variable was observed while keeping all the other variables constant at their average value. A detailed description of the parametric analysis results is given in the following sections.

##### Geometric Parameters

**Figure 6:**
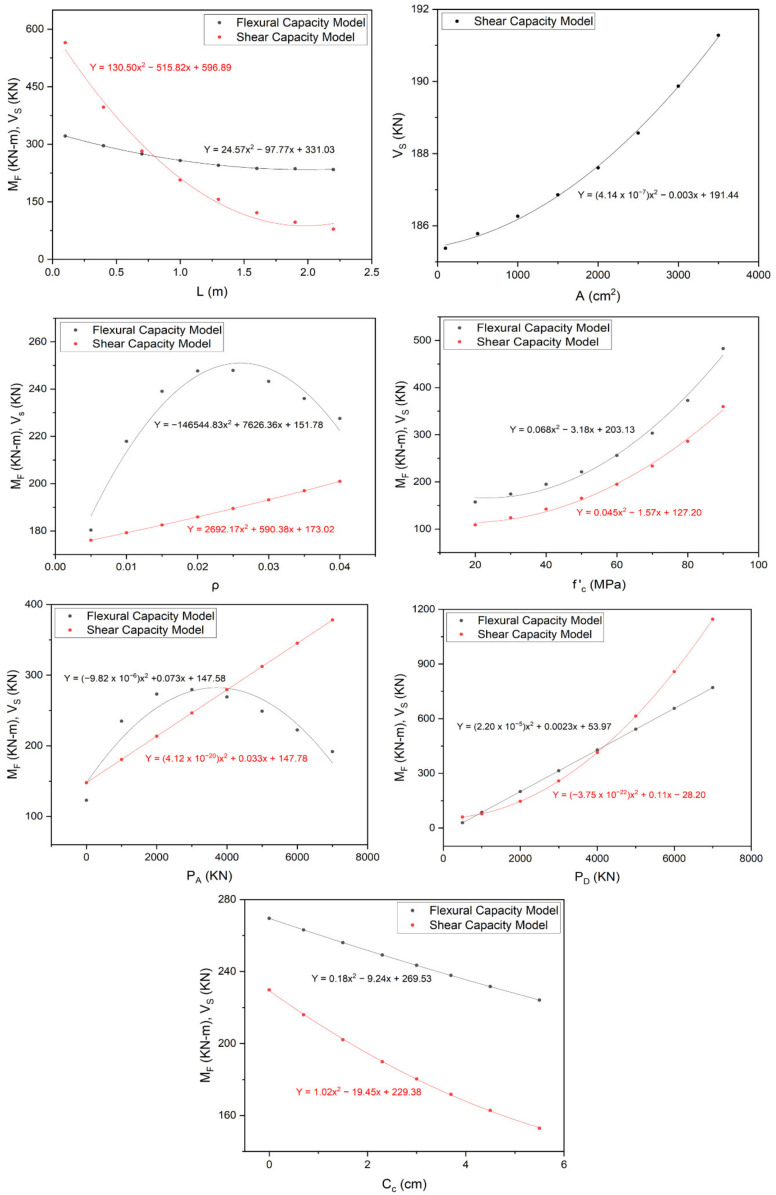
Parametric Analysis Results for Proposed Capacity Prediction Models.

Two geometric parameters, i.e., *L* and *A* were selected as the input variables for developing GEP models for predicting the bearing capacity of RC rectangular columns. The impact of these variables on the overall bearing capacity of the aforementioned columns was investigated based on parametric analysis, the results of which are shown in Figure 6. The figure shows an increase in the flexural and shear capacity of RC rectangular columns along with the decrease in length, and the increase in the cross-sectional area of columns. The inverse relationship between the length and flexural or shear capacity of the RC rectangular columns is because of the probability of buckling phenomenon in the long columns. Similar results were also presented in the nonlinear finite element-based study conducted by Mahmood and Ghulam [14], which ensures the strength and effectiveness of the proposed models.

##### Material Parameters

*f ‘_c_* was the only material characteristic considered in developing the proposed models. Based on the parametric analysis, its impact on the overall bearing capacity of RC rectangular columns is presented in Figure 6. This figure shows a direct relationship between the flexural and shear capacity of RC rectangular columns and *f ‘_c_*. A similar relationship was also observed by the finite element-based research program of Mahmood and Ghulam [14]. This relationship can also be validated by Equation (18) of ACI 318-19 [87].

##### Structural Parameters

Four structural parameters of RC rectangular columns, i.e., *ρ*, *P_A_*, *P_D_*, and *C_c_*, were considered in developing their capacity prediction GEP models. The impact of these parameters on the overall bearing capacity of RC rectangular columns was analyzed based on parametric analysis, the results of which are presented in Figure 6. The figure shows that the flexural and shear capacity of the RC rectangular column increases along the increase in *ρ*, *P_A_*, and *P_D_*, whereas it decreases along with the increase in *C_c_*. Moreover, flexural capacity was found to be declining after reaching a certain threshold of *ρ* and *P_A_*. These observations were found to be well in agreement with the non-linear finite element-based investigations of Mahmood and Ghulam [14] and the design guidelines of ACI 318-19 [87]. The trend of predicted output corresponding to a certain change in these parameters was also found to be consistent with the experimental results in the database which further authenticates the validity of the proposed models.

### 3.4. Performance Evaluation

**Figure 7:**
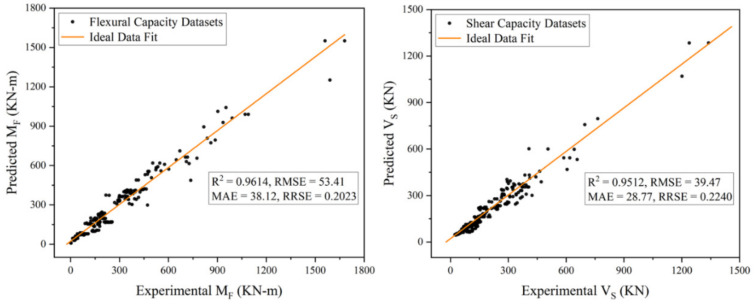
Predicted Versus Experimental Values Plot for Proposed Models.

**Figure 8:**
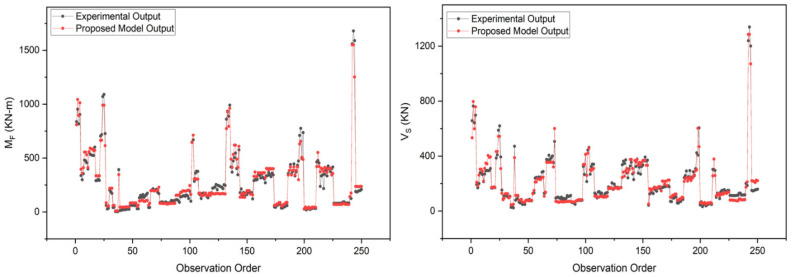
Comparison of Experimental and Proposed Model Results.

**Figure 9:**
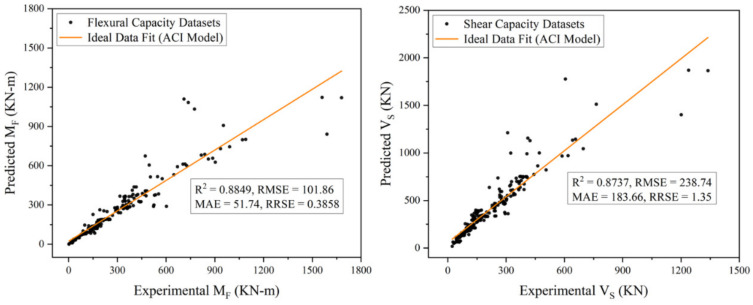
Predicted Versus Experimental Values Plot for ACI Models.

The performance of both the proposed capacity prediction models was evaluated based on four key fitness indicators, i.e., *R^2^*, *RMSE*, *MAE*, and *RRSE,* as described earlier in the “GEP Modelling” section. For this evaluation, some fundamental statistical analyses were employed, which reported these performance indicators to be 0.9614, 53.41, 38.12, and 0.2023, respectively, for the flexural capacity model, and 0.9512, 39.47, 28.77, and 0.2240, respectively, for the shear capacity model (The details of these evaluation results are shown in Figure 7). From these performance indicators, it can be observed that there exists a strong correlation between the predicted and experimental output with the least possible error (Figure 8), which indicates the higher prediction accuracy of the proposed models. In addition to these fitness criteria, the performance of the proposed models was also assessed by comparing with the prevailing design code of concrete structures in the country (Pakistan) i.e., ACI 318-19 [87]. (Explanation: The comparison was made only with ACI 318-19 [87] because almost all the design codes provide similar guidelines for the capacity prediction of discussed RC members. Moreover, no specific mathematical expressions were found by the author in past research articles, on the basis of which comparison could have been made.) For the comparison, the flexural and shear strength of RC rectangular columns present in the database were computed in accordance with the guidelines of sections 22.4 and 22.5 of ACI 318-19 [87], the mathematical expressions for which are also given in Equations (17)–(20). All the parameters involved in these equations are defined clearly in Chapter 2 of the given design code. The performance of ACI model was also evaluated by employing the same statistical analysis. The results of these analyses reported *R^2^*, *RMSE*, *MAE*, and *RRSE* to be 0.8849, 101.86, 51.74, and 0.3858, respectively, for flexural capacity predictions, whereas 0.8737, 238.74, 183.66, and 1.35 respectively for shear capacity predictions (Figure 9). The comparison depicted a better performance and higher accuracy of the proposed models than that of ACI 318-19 [87]. Following the model validation and performance evaluation results, given models can be recommended for computing the lateral load-carrying capacity of RC rectangular columns for future practical purposes.

(17) $$V_{S}=V_{c}+V_{st},$$

(18) $$V_{c}=\left[0.66\lambda {\left(\rho_{w}\right)}^{\frac{1}{3}}\sqrt{{f^{\prime }}_{c}}+\frac{N_{u}}{6A_{g}}\right]b_{w}d,$$

(19) $$V_{st}=\frac{A_{v}f_{yt}d}{S},$$

(20) $$V_{S}=\frac{2M_{F}}{L},$$

## 4. Conclusions

This research presented a novel AI approach to GEP for predicting the ultimate lateral load-carrying capacity of RC rectangular columns subjected to earthquake loading. For this, an experimental database assembled by the PEER centre consisting of 250 cyclic tests of RC rectangular columns was utilized. Based on statistical GEP modelling results as presented in the earlier sections, the following conclusions can be drawn:

- The proposed AI technique provides an alternative method for the determination of lateral load carrying capacity of RC rectangular columns while avoiding complicated structural and mathematical computations. Moreover, it is also simpler and easier to be implemented in practical applications.
- The proposed capacity prediction models were found to exhibit better accuracy when compared to that of the ACI model. The major performance indicator, i.e., *R*^2^, was found to be 0.9614 and 0.9512 in the proposed flexural and shear capacity model, respectively, and 0.8849 and 0.8737 in the case of flexural and shear capacity models ACI, respectively.
- Design axial load (*P_D_*) was found to be the most significant input variable, contributing around 50% towards the development of both the proposed models. The rest of the six input variables were observed to cumulatively account for the remaining 50% of the overall model development.
- From the parametric analysis results of the proposed models, the trend of output variables corresponding to most of the input variables was found to be consistent with the experimental results in the database, which validates the ability of the proposed models to capture behind the scenes real-world phenomena.

The main incentive of the proposed models is that they are based on a “black box” model that relates the inputs and outputs in a simplified manner while avoiding complex mathematical derivations. It has better performance as compared to the traditional analytical models and therefore can be employed to predict the lateral load carrying capacity of RC rectangular columns quickly, accurately, and robustly. Moreover, the proposed GEP models can be expressed in terms of simple mathematical expressions, which is not possible in some of the other conventional and modern analytical techniques. Apart from all its advantages, it is associated with certain limitations. The proposed models are unable to explain the failure mode, as well as the involved mechanism in predicting the lateral load carrying capacity of RC rectangular columns. Moreover, as it was not feasible to consider every involved parameter in the model development, the applicability of the proposed models in special cases is questionable. Considering all the pros and cons of the proposed capacity prediction models, future researchers are recommended to work on the best AI algorithm, either individual or ensembled [88,89,90,91,92,93,94,95,96], that considers all the possible aspects and explains the mechanism involved.

## Figures and Tables

**Table 1:** Database Characteristics.

Property	Unit	Statistical Parameters		
		Mean	STD	COV
Depth	mm	319	117	0.37
Aspect Ratio	Decimals	3.58	1.46	0.41
Axial Load Ratio	Decimals	0.27	0.19	0.70
Longitudinal Reinforcement Ratio	%	2.39	0.96	0.40
Transverse Reinforcement Ratio	%	2.01	1.22	0.61

STD: Standard Deviation, COV: Coefficient of Variations.

**Table 2:** Parametric Selection Based on Linear Regression and Cosine Amplitude.

Parameters	Flexural Capacity Output		Shear Capacity Output	
	*p*-Value	*R*-Value	*p*-Value	*R*-Value
Column Length	0.2209	0.8101	1.04 × 10^−44^	0.7199
Cross Sectional Area	6.72 × 10^−12^	0.9342	1.59 × 10^−11^	0.8889
Long. Rein. Ratio	0.0005	0.6239	0.0102	0.6985
Long. Rein. Yield Strength	0.4242	0.6818	0.5124	0.7348
Long. Rein. Ultimate Strength	0.8822	0.6417	0.2295	0.6391
Trans. Rein. Ratio	0.1707	0.5013	0.1874	0.5181
Trans. Rein. Yield Strength	0.7121	0.5861	0.0921	0.6881
Trans. Rein. Ultimate Strength	0.7703	0.5797	0.0995	0.6186
Concrete Compressive Strength	0.0083	0.5741	0.0718	0.6585
Applied Axial Load	9.25 × 10^−18^	0.8053	3.64 × 10^−21^	0.7979
Design Axial Load	0.0081	0.9068	9.63 × 10^−05^	0.8839
Clear Cover	0.0027	0.7468	0.3666	0.7707

Long.: Longitudinal, Rein.: Reinforcement, Trans.: Transverse.

**Table 3:** Statistical Characteristics of Model Parameters.

Parameters	Symbol	Unit	Type	Minimum	Maximum	Mean	STD
Column Length	*L*	m	Input	0.08	2.34	1.095	0.5485
Cross Sectional Area	*A*	cm^2^	Input	64	4180.64	1021.6	777.88
Long. Rein. Ratio	*ρ*	Decimal	Input	0.007	0.0603	0.024	0.0101
Concrete Comp. Strength	*f ’_c_*	MPa	Input	16	118	51.91	29.244
Applied Axial Load	*P_A_*	KN	Input	0	8000	1238.33	1350.28
Design Axial Load	*P_D_*	KN	Input	109.51	7359.6	2424.26	1421.61
Clear Cover	*C_c_*	cm	Input	0	6.51	2.395	1.0855
Flexural Capacity	*M_F_*	KN-m	Output	2	1680	264.75	264.55
Shear Capacity	*V_S_*	KN	Output	23	1339	207.78	176.55

Comp.: Compressive, *P_D_*: Function of Geometric and Material Properties.

**Table 4:** Summary of GEP Models for Predicting the Bearing Capacity of RC Rectangular Columns.

GEP Models	Model Details								Performance Indicators			
	Chromosomes	Head Size	Genes	Linking Function	Fitness Function	Model Functions	Input Variables	Variables Used	*R* ^2^	*RMSE*	*MAE*	*RRSE*
Flexural Capacity Models (FCM)												
FCM 1	30	8	3	+	*RMSE*	+, −, *, /	7	7	0.9228	73.42	47.21	0.2781
FCM 2	30	8	3	−	*RMSE*	+, −, *, /	7	5	0.9275	71.10	48.03	0.2693
FCM 3	30	8	3	*	*RMSE*	+, −, *, /	7	6	0.9448	62.17	41.82	0.2355
FCM 4	30	8	3	/	*RMSE*	+, −, *, /	7	7	0.9454	61.94	45.67	0.2346
FCM 5	30	8	3	Average	*RMSE*	+, −, *, /	7	7	0.9233	74.09	47.03	0.2806
FCM 6	30	8	3	Minimum	*RMSE*	+, −, *, /	7	7	0.9221	74.33	46.89	0.2815
FCM 7	30	8	3	Maximum	*RMSE*	+, −, *, /	7	6	0.9156	88.17	60.35	0.3340
FCM 8	80	8	3	/	*RMSE*	+, −, *, /	7	7	0.9496	59.68	41.90	0.2260
FCM 9	50	8	3	/	*RMSE*	+, −, *, /	7	7	0.9614	53.41	38.12	0.2023
FCM 10	50	12	4	/	*RMSE*	+, −, *, /	7	7	0.9376	66.01	42.78	0.2500
FCM 11	50	5	2	/	*RMSE*	+, −, *, /	7	5	0.9298	70.13	43.56	0.2656
FCM 12	50	8	3	/	*RMSE*	+, −, *, /, √	7	6	0.9362	66.97	45.06	0.2536
FCM 13	50	8	3	/	*RMSE*	+, −, *, /, ln	7	7	0.9264	71.63	43.96	0.2713
Shear Capacity Models (SCM)												
SCM 1	30	8	3	+	*RMSE*	+, −, *, /	7	6	0.9233	48.81	32.94	0.2770
SCM 2	30	8	3	−	*RMSE*	+, −, *, /	7	7	0.9012	55.40	39.05	0.3144
SCM 3	30	8	3	*	*RMSE*	+, −, *, /	7	6	0.9138	51.80	37.88	0.2940
SCM 4	30	8	3	/	*RMSE*	+, −, *, /	7	6	0.9246	49.11	35.69	0.2787
SCM 5	30	8	3	Average	*RMSE*	+, −, *, /	7	7	0.9032	56.41	38.40	0.3201
SCM 6	30	8	3	Minimum	*RMSE*	+, −, *, /	7	7	0.9133	53.36	40.25	0.3029
SCM 7	30	8	3	Maximum	*RMSE*	+, −, *, /	7	7	0.9234	48.88	35.24	0.2774
SCM 8	80	8	3	/	*RMSE*	+, −, *, /	7	6	0.9268	47.71	35.05	0.2708
SCM 9	50	8	3	/	*RMSE*	+, −, *, /	7	7	0.9481	40.23	29.13	0.2283
SCM 10	50	12	4	/	*RMSE*	+, −, *, /	7	7	0.9052	54.30	33.45	0.3082
SCM 11	50	5	2	/	*RMSE*	+, −, *, /	7	5	0.8942	58.46	44.75	0.3318
SCM 12	50	8	3	/	*RMSE*	+, −, *	7	7	0.9512	39.47	28.77	0.2240
SCM 13	50	8	3	/	RMSE	+, −, *, /, √	7	5	0.9287	47.21	34.01	0.2679

## Data Availability

The data presented in this study can be obtained on request from the corresponding author.
